# 1183. Serum Bactericidal Activity Induced by Live Attenuated Pertussis Vaccine BPZE1 is Comparable to Boostrix™

**DOI:** 10.1093/ofid/ofab466.1375

**Published:** 2021-12-04

**Authors:** Cheryl A Keech, Andrew Gorringe, Breeze Cavell, Peter Goldstein, Keith Rubin

**Affiliations:** 1 ILiAD Biotechnologies, Weston, Florida; 2 Public Health England, Portland, England, United Kingdom

## Abstract

**Background:**

In a Phase 2b, multi-center, placebo-controlled, randomized study, intranasal BPZE1 induced mucosal and serum antibodies to pertussis antigens and protected against subsequent colonization following attenuated challenge with BPZE1 3 months later. Boostrix^TM^ also induced serum but not mucosal antibodies and did not protect against BPZE1 challenge. We have evaluated the induction of serum bactericidal activity (SBA) for *Bordetella pertussis* by BPZE1 or Boostrix vaccination. A previous study showed that Boostrix induction of SBA is dependent on Prn whereas *B. pertussis* infection induces SBA targeting Prn and other antigens.

**Methods:**

A convenience set of subjects who had a broad range of Prn and PT IgG serum concentrations from treatment groups who received BPZE1+BPZE1 or Boostrix+Placebo (Day 1 and 85 vaccination) were randomly selected to assess SBA using *B. pertussis* strain B1917. Three timepoints (baseline, 28 days following first and second vaccination) were analyzed and interpolated 50% killing titers determined. The relationship to Prn IgG concentration was assessed.

**Results:**

BPZE1 and Boostrix elicited similar and significant increases in SBA following vaccination. BPZE1 and Boostrix elicit anti-Prn IgG, with Boostrix eliciting higher concentrations. A greater SBA response relative to PRN IgG was observed for BPZE1 compared to Boostrix. SBA-Prn correlations were high post-Boostrix (0.74) as previously reported; correlation was lower (0.35) following BPZE1, suggesting the involvement of broader antigenic protection beyond Prn alone.

Table of GMT and GMFR in SBA and Prn IgG

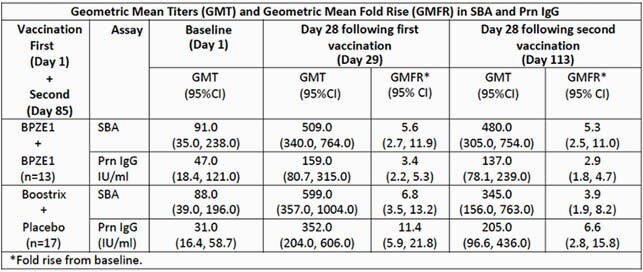

**Conclusion:**

In this exploratory investigation, the novel intranasal live-attenuated pertussis vaccine BPZE1 induced SBA titers that were similar to Boostrix using a *B. pertussis* strain representative of current disease isolates. SBA-Prn correlations were high post-Boostrix, consistent with prior reports showing Prn is the acellular vaccine antigen that mediates SBA. In contrast, BPZE1 bactericidal antibodies appear broader than Prn which may be important given the global rise of Prn-deficient *B. pertussis* strains.

**Disclosures:**

**All Authors**: No reported disclosures

